# Identification and multi-layered validation of seven diagnostic biomarkers for dilated cardiomyopathy via integrative machine learning, single-cell transcriptomics, and Mendelian randomization

**DOI:** 10.3389/fcell.2026.1851275

**Published:** 2026-06-09

**Authors:** Jingwei Li, Zhongyang Song, Guanwei Wang, Yuchan Chen, Jiamiao Cheng, Zhiming Zhang

**Affiliations:** 1 College of Clinical Traditional Chinese Medicine, Gansu University of Chinese Medicine, Lanzhou, China; 2 Department of Oncology, Affiliated Hospital of Gansu University of Traditional Chinese Medicine, Lanzhou, China; 3 Gansu Institute of Cardiovascular Diseases, Lanzhou, China; 4 College of Integrated Traditional Chinese and Western Medicine, Gansu University of Chinese Medicine, Lanzhou, China; 5 Gansu Provincial Hospital of Traditional Chinese Medicine, Lanzhou, China

**Keywords:** co-expression-based functional importance score, diagnostic biomarkers, dilated cardiomyopathy, immune microenvironment, machine learning, Mendelian randomization, single-cell RNA sequencing, WGCNA

## Abstract

**Background:**

Dilated cardiomyopathy (DCM) is the most common non-ischemic cardiomyopathy and a major cause of heart failure, but disease-specific molecular biomarkers remain limited. This study aimed to identify and prioritize tissue-level, disease-responsive candidate biomarkers for DCM using an integrative multi-omics bioinformatics framework.

**Methods:**

Bulk myocardial transcriptomic data from GSE57338 were used as the discovery cohort, and GSE26887, GSE42955, and GSE79962 served as external microarray validation cohorts. GSE116250 was used for independent RNA-seq validation. Differentially expressed genes were intersected with WGCNA hub genes to define candidate genes. Four machine-learning algorithms, including LASSO, random forest, SVM-RFE, and XGBoost, were applied to identify core diagnostic candidates. Tissue-level model performance was evaluated by ROC analysis, calibration assessment, nomogram visualization, and decision curve analysis. Orthogonal validation was performed using GTEx, HPA, and snRNA-seq data. Immune infiltration, bidirectional Mendelian randomization, and CellOracle-based GRN analysis with a co-expression-based functional importance score were used as hypothesis-generating analyses. The workflow explicitly separated diagnostic performance, localization evidence, and exploratory mechanistic context in myocardial tissue.

**Results:**

Integration of 309 DEGs and 2,093 WGCNA hub genes yielded 270 candidates. Seven candidates—HMGN2, AQP3, SERPINA3, FREM1, HMOX2, CSDC2, and TUBA3E—were selected by at least three algorithms. In the discovery cohort, the RF model achieved an AUC of 0.985 and the logistic model achieved a C-statistic of 0.993; however, these estimates were interpreted as potentially optimistic upper bounds because feature selection was not nested within cross-validation. External validation showed uneven robustness: SERPINA3, HMOX2, FREM1, and HMGN2 were consistently supported across microarray and RNA-seq cohorts, whereas AQP3, CSDC2, and TUBA3E were exploratory. GTEx, HPA, and snRNA-seq supported cardiac expression and cell-type localization, including cardiomyocyte enrichment of CSDC2/HMOX2 and fibroblast enrichment of FREM1. MR and GRN analyses suggested disease-responsive rather than disease-driving biology, including possible heart failure-associated AQP3 downregulation and a putative PPARGC1A–CSDC2/HMOX2 regulatory context.

**Conclusion:**

This study identifies seven prioritized, predominantly disease-responsive tissue-level molecular candidates for DCM. These findings provide candidates and testable hypotheses for future translational research, rather than disease-driving therapeutic targets or a directly applicable clinical test.

## 1 Introduction

Dilated cardiomyopathy (DCM) is a primary myocardial disorder characterized by left ventricular or biventricular dilation and systolic dysfunction in the absence of coronary artery disease or abnormal loading conditions sufficient to explain the degree of impairment (Knowlton, 2019; McNally and Mestroni, 2017). As the most common form of non-ischemic cardiomyopathy, DCM accounts for approximately 30%–40% of heart failure cases and remains a leading indication for cardiac transplantation worldwide (Hershberger et al., 2013; Savarese and Lund, 2017). Despite advances in pharmacological and device-based therapies, including neurohormonal blockade and cardiac resynchronization therapy, the 5-year mortality rate of patients with DCM remains substantial at 20%–30% (Merlo et al., 2018; Bozkurt et al., 2016). Early and accurate identification of DCM is therefore critical for timely intervention and improved patient outcomes.

Current diagnostic strategies for DCM rely predominantly on imaging modalities, including echocardiography and cardiac magnetic resonance imaging, supplemented by circulating biomarkers such as B-type natriuretic peptide (BNP), N-terminal pro-BNP, and cardiac troponins (Japp et al., 2016; Ponikowski et al., 2016). However, these conventional biomarkers lack specificity for DCM, as they are elevated across a broad spectrum of cardiac conditions, including ischemic heart disease, hypertensive heart disease, and valvular disorders (Seferović et al., 2019). Moreover, endomyocardial biopsy—the reference standard for myocardial tissue characterization—is invasive, carries procedural risks, and is subject to sampling error (Schultheiss et al., 2019). Therefore, there remains a need to identify candidate molecular biomarkers that can improve tissue-level characterization of DCM and may ultimately inform future non-invasive diagnostic strategies.

The rapid expansion of publicly available transcriptomic repositories, particularly the Gene Expression Omnibus (GEO) database, has created new opportunities for systematic biomarker discovery (Barrett et al., 2013). Integrative bioinformatics approaches that combine differential expression analysis with network-based methods such as weighted gene co-expression network analysis (WGCNA) can reduce the dimensionality of high-throughput data while preserving biologically meaningful gene modules associated with disease phenotypes (Langfelder and Horvath, 2008; Zhang and Horvath, 2005). Furthermore, applying multiple complementary machine learning algorithms—including LASSO regression, random forests, support vector machines, and gradient boosting—within a convergence framework may improve feature prioritization by reducing dependence on any single method (Saeys et al., 2007; Zou and Hastie, 2005).

Beyond gene identification, understanding the biological context of candidate biomarkers is important for prioritizing signals for further investigation. The immune microenvironment plays a well-established role in DCM pathogenesis, with both innate and adaptive immune responses contributing to myocardial inflammation, fibrosis, and adverse ventricular remodeling (Heymans et al., 2009; Epelman et al., 2015). Mendelian randomization (MR) can provide useful evidence for distinguishing potential causal relationships from associations by leveraging genetic variants as instrumental variables (Davey Smith and Hemani, 2014; Burgess et al., 2013). In addition, large-scale expression quantitative trait loci (eQTL) resources, including the eQTLGen consortium and the Genotype-Tissue Expression (GTEx) project, allow blood-derived and tissue-specific genetic instruments to be used for exploratory assessment of whether candidate biomarkers may reflect disease-driving or disease-responsive processes (Võsa et al., 2021; GTEx Consortium, 2020).

In the present study, we developed a multi-layer bioinformatic discovery and prioritization pipeline for DCM candidate biomarkers, organized around three levels of evidence with distinct interpretive strength. Primary evidence (Tier 1) included differential expression analysis, WGCNA, four-algorithm machine-learning feature selection, and external validation of tissue-level diagnostic performance across three independent microarray cohorts and an independent RNA-seq cohort (GSE116250). Orthogonal localization evidence (Tier 2) integrated GTEx multi-tissue expression, HPA immunohistochemistry, and single-nucleus transcriptomics from human DCM hearts to assess tissue specificity, RNA–protein concordance, and cell-type-resolved expression (Chaffin et al., 2022; Uhlén et al., 2015; Litviňuková et al., 2020). Hypothesis-generating mechanistic context (Tier 3) included immune microenvironment analysis, bidirectional MR using blood-derived and cardiac tissue-specific eQTLs, and CellOracle-based GRN analysis with co-expression-based functional importance scoring. Because the MR analyses did not identify significant forward causal effects, the prioritized genes are interpreted primarily as tissue-level disease-responsive candidates rather than confirmed disease-driving genes or therapeutic targets. Throughout the manuscript, we interpret model performance as evidence of myocardial tissue-level discrimination rather than direct clinical applicability, and we treat the mechanistic analyses as hypothesis-generating rather than causal. To our knowledge, this study provides a comprehensive bioinformatic discovery framework for prioritizing molecular candidates for future experimental and translational investigation in DCM.

## 2 Materials and methods

### 2.1 Data acquisition and preprocessing

Gene expression datasets were retrieved from the NCBI Gene Expression Omnibus (GEO) database (Barrett et al., 2013). The discovery cohort, GSE57338 (GPL11532, Affymetrix HuGene-1.1-ST), comprised 218 left ventricular myocardial tissue samples (82 DCM and 136 non-failing controls) (Liu et al., 2015). Three independent external validation cohorts were obtained: GSE26887 (n = 24; 19 heart failure vs. 5 controls; GPL6244) (Molina-Navarro et al., 2013), GSE42955 (n = 17; 12 DCM vs. 5 normal; GPL6244) (Barth et al., 2011), and GSE79962 (n = 20; 9 DCM vs. 11 donors; GPL6244) (Tarazón et al., 2014). An additional RNA-seq cohort, GSE116250 (Illumina HiSeq 2500; 37 DCM vs. 14 non-failing left ventricular samples), was included to assess cross-platform generalizability (Sweet et al., 2018). All microarray expression matrices were log2-transformed and quantile-normalized; GSE116250 RPKM values were log2 (RPKM + 1)-transformed. Probe-to-gene mapping was performed using platform annotation files, with duplicate probes resolved by retaining the probe with the highest mean expression. After quality control, 18,615 protein-coding genes were retained in the discovery set. The characteristics of all datasets used in this study, including the RNA-seq validation cohort, are summarized in Table 1.

**TABLE 1:** Characteristics of GEO datasets used in this study.

Dataset	Platform	Tissue	DCM (n)	Control (n)	Total (n)	Role
GSE57338	GPL11532 (HuGene-1.1-ST)	Left ventricle	82	136	218	Discovery
GSE26887	GPL6244 (HuGene-1.0-ST)	Left ventricle	19	5	24	Validation
GSE42955	GPL6244 (HuGene-1.0-ST)	Left ventricle	12	5	17	Validation
GSE79962	GPL6244 (HuGene-1.0-ST)	Left ventricle	9	11	20	Validation
GSE116250	Illumina HiSeq 2500/RNA-seq	Left ventricle	37	14	51	RNA-seq validation

DCM, dilated cardiomyopathy. The discovery cohort (GSE57338) was used for differential expression analysis, WGCNA, machine-learning feature selection, and model training. Three microarray cohorts (GSE26887, GSE42955, GSE79962) and one RNA-seq, cohort (GSE116250) served as external validation datasets. GSE116250 originally included ischemic cardiomyopathy samples, but only DCM, and non-failing samples were used in this study.

### 2.2 Differential expression analysis

Differential expression analysis between DCM and control samples in the discovery cohort was performed using the limma package with empirical Bayes moderation (Ritchie et al., 2015). Genes with a Benjamini–Hochberg adjusted P-value < 0.05 and an absolute log_2_ fold change > 0.585, corresponding to a fold change > 1.5, were defined as differentially expressed genes (DEGs).

### 2.3 Weighted gene co-expression network analysis

WGCNA was performed using the WGCNA R package on the top 5,000 most variable genes, ranked by median absolute deviation, across all 218 samples in the discovery cohort (Langfelder and Horvath, 2008). A signed network was constructed using a soft-thresholding power of 13, which achieved scale-free topology fit (*R* ^2^ > 0.80), with a minimum module size of 30 and a merge cut height of 0.25. Module eigengenes were correlated with DCM status using Pearson correlation. Hub genes within significantly associated modules (|r| > 0.3, P < 0.05) were identified using module membership (MM > 0.5) and gene significance (GS > 0.15). The intersection of DEGs and WGCNA hub genes defined the candidate gene set for downstream machine-learning analysis.

### 2.4 Machine learning-based feature selection

Four complementary algorithms were applied to prioritize features from the candidate gene set: (1) LASSO logistic regression with 10-fold cross-validation using the glmnet package (Tibshirani, 1996); (2) random forest with 500 trees, selecting the top 30 genes by mean decrease in Gini impurity (Breiman, 2001); (3) SVM-RFE with a linear kernel, selecting the top 30 genes by elimination ranking (Guyon et al., 2002); and (4) XGBoost gradient boosting, selecting the top 30 genes by gain-based importance (Chen and Guestrin, 2016). Genes supported by at least three of the four algorithms were defined as core diagnostic candidates for downstream tissue-level model evaluation.

### 2.5 Diagnostic model construction and evaluation

Diagnostic performance was evaluated using ROC curve analysis with the pROC package (Robin et al., 2011). In the discovery cohort, 10 repeats of 10-fold cross-validation were used to evaluate four classifiers: logistic regression, LASSO, SVM, and RF. A multivariate logistic regression model was constructed using the rms package (Harrell, 2001) and visualized with a nomogram. Calibration was assessed using 200 bootstrap resamples in the discovery cohort and binned calibration in the validation cohorts. Decision curve analysis quantified the analytical net benefit of the tissue-based classifier across threshold probabilities (Vickers and Elkin, 2006), which was interpreted as model performance across decision cutoffs rather than direct evidence of clinical utility.

#### 2.5.1 Sequence of feature selection and model evaluation

Differential expression analysis, WGCNA hub-gene extraction, and four-algorithm machine-learning convergence were performed once using the full GSE57338 discovery cohort. The resulting seven-gene panel was then evaluated by repeated 10-fold cross-validation. Therefore, feature selection was not nested within each cross-validation fold, and the discovery-set AUC, C-statistic, and calibration estimates may be optimistic. We therefore interpret the discovery-set metrics as upper-bound estimates and place greater emphasis on the independent external cohorts—GSE26887, GSE42955, GSE79962, and GSE116250—which were not used for feature selection, model fitting, or hyperparameter tuning.

### 2.6 Leave-one-gene-out analysis

To estimate each gene’s contribution to the tissue-level diagnostic model, leave-one-gene-out (LOOGO) analysis was performed. For each of the seven core diagnostic candidates, a reduced six-gene logistic regression model was trained in the discovery cohort, and the AUC was calculated in both the discovery and validation cohorts. The change in AUC (ΔAUC = AUC_full − AUC_reduced) was used to assess the relative contribution of each gene to model performance.

### 2.7 Functional enrichment analysis

Gene Ontology (GO) biological process and Kyoto Encyclopedia of Genes and Genomes (KEGG) pathway enrichment analyses were performed on the 309 DEGs identified in the discovery cohort using clusterProfiler (Yu et al., 2012; Ashburner et al., 2000; Kanehisa and Goto, 2000). Gene set enrichment analysis (GSEA) was conducted on the complete ranked gene list against KEGG collections (Subramanian et al., 2005).

### 2.8 Immune infiltration analysis

The relative abundance of 16 immune cell types was quantified using ssGSEA via the GSVA package (Hänzelmann et al., 2013), based on immune cell gene signatures from a published compendium (Charoentong et al., 2017). Group differences were assessed using Wilcoxon rank-sum tests with Benjamini–Hochberg correction. Spearman correlations between core diagnostic candidate expression and immune cell abundance were computed. As a sensitivity analysis, immune cell abundance was independently estimated using curated marker-gene scores based on canonical cell-type markers, such as CD68/CD163 for macrophages and NKG7/GNLY for NK cells, and directional concordance with ssGSEA results was assessed.

### 2.9 GTEx multi-tissue expression validation

To assess the tissue expression landscape of the core diagnostic candidates, median gene-level transcripts per million (TPM) values across 54 human tissues were obtained from the GTEx v8 dataset (n = 948 donors) (GTEx Consortium, 2020). Expression values were log2 (TPM + 1)-transformed. Heatmap visualization was used to evaluate cardiac tissue expression relative to other organs.

### 2.10 Human protein Atlas protein expression validation

Protein-level expression data were obtained from the Human Protein Atlas (HPA; version 23) (Uhlén et al., 2015). Immunohistochemistry (IHC) staining results for heart muscle were extracted from the normal tissue dataset, and protein detection levels were classified as High, Medium, Low, or Not detected. Consensus RNA expression values (nTPM) were also retrieved to assess RNA–protein concordance.

### 2.11 Single-nucleus RNA sequencing validation

Published snRNA-seq data from human left ventricular tissue (GSE183852) were obtained from the Human Cell Atlas Data Explorer (Chaffin et al., 2022). This dataset comprised 592,689 nuclei from 11 DCM, 15 hypertrophic cardiomyopathy (HCM), and 16 non-failing (NF) hearts. For this study, only DCM and NF samples were retained (n = 357,437 nuclei), followed by random subsampling to 80,000 nuclei to reduce computational burden. Data were processed using Scanpy (v1.12) (Wolf et al., 2018). Downsampling was performed with a fixed random seed (seed = 42), and stability was assessed using two additional seeds (123 and 456), confirming high reproducibility (Spearman r > 0.96 across pairwise comparisons). After normalization (target sum = 10,000) and log transformation, the expression of the seven core diagnostic candidates was visualized across 21 annotated cell types using UMAP embeddings, dot plots, and violin plots. Differential expression between DCM and NF conditions was assessed within cardiomyocyte populations.

### 2.12 Bidirectional Mendelian randomization (blood eQTL)

Two-sample MR was performed using the TwoSampleMR package (Hemani et al., 2018). For the forward direction (gene expression → heart failure), cis-eQTL variants were obtained from the eQTLGen consortium (n = 31,684) (Võsa et al., 2021). Three GWAS outcomes were tested: HERMES heart failure (ebi-a-GCST009541) (Shah et al., 2020), FinnGen heart failure (finn-b-I9_HEARTFAIL) (Kurki et al., 2023), and FinnGen cardiomyopathy (finn-b-I9_CARDMYO). The Wald ratio method was used for single-SNP instruments, and inverse-variance weighted analysis was used when ≥2 SNPs were available, with MR-Egger and weighted median methods applied as sensitivity analyses. For the reverse direction (heart failure → gene expression), GWAS instruments from HERMES, FinnGen, and EBI chronic heart failure (ebi-a-GCST90018806) (Sakaue et al., 2021) were used with eQTLGen eQTLs as outcomes. Instruments with F-statistics > 10 were considered to have adequate strength.

### 2.13 Cardiac tissue-specific Mendelian randomization

To address the limited tissue specificity of blood-based eQTLs, we repeated the forward MR analysis using heart left ventricle-specific eQTLs from GTEx v8 (n = 386 samples) (GTEx Consortium, 2020). Lead eQTL variants were extracted for each core diagnostic candidate, formatted as exposure instruments, and tested against the same GWAS outcomes. Results were compared with blood eQTL-based findings to explore tissue-specific concordance.

### 2.14 Gene regulatory network inference and co-expression analysis

Gene regulatory network (GRN) inference was performed using CellOracle (v0.20.0) (Kamimoto et al., 2023) within the WSL (Windows Subsystem for Linux) environment. After subsampling the snRNA-seq data to 12,000 nuclei and selecting 2,506 highly variable genes, including all seven core diagnostic candidates, KNN imputation and ridge regression-based GRN fitting were conducted across 21 cell-type clusters using the human promoter base GRN (hg19_gimmemotifsv5_fpr2). Because the seven core diagnostic candidates are not transcription factors (TFs) and therefore cannot be directly used as perturbation inputs in CellOracle’s TF-centered simulation framework, we identified putative upstream TF regulators by computing Spearman correlations between TF expression and candidate gene expression across the snRNA-seq data. In addition, a co-expression-based functional importance score was calculated for each gene–cell type pair as the product of mean expression, fraction of expressing cells, and summed absolute co-expression with the remaining core candidates. This author-defined score was used only as a descriptive proxy to prioritize gene–cell type pairs with potentially greater transcriptional relevance. It was not benchmarked against CRISPR screens, RNAi experiments, or CellOracle perturbation outputs, and should not be interpreted as a validated prediction of knockout, perturbation, or gene-ablation effects. Protein–protein interaction (PPI) data were retrieved from the STRING database (v12.0) (Szklarczyk et al., 2023).

### 2.15 Statistical analysis

All analyses were performed in R (v4.5.3) and Python (v3.12). Continuous variables were compared using Student’s t-test or Wilcoxon rank-sum test, as appropriate. Correlations were assessed using Spearman’s rank correlation. Multiple testing correction was performed using the Benjamini–Hochberg method where applicable. Two-sided P < 0.05 was considered statistically significant unless otherwise specified.

## 3 Results

**FIGURE 1:**
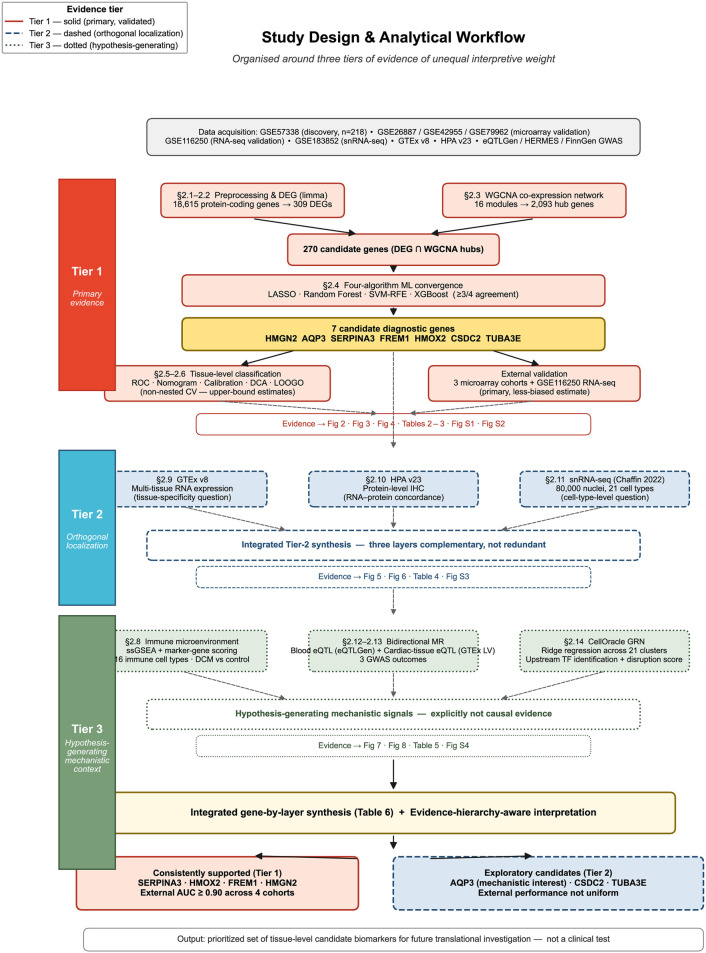
Study workflow organized around three tiers of evidence. The workflow illustrates the multi-layer bioinformatic discovery and prioritization pipeline used in this study. Tier 1 includes data acquisition, differential expression analysis, WGCNA, four-algorithm machine-learning feature selection, tissue-level model construction, and external validation in microarray and RNA-seq cohorts. Tier 2 includes orthogonal localization evidence from GTEx tissue expression, HPA immunohistochemistry, and snRNA-seq cell-type localization. Tier 3 includes hypothesis-generating mechanistic context from immune profiling, bidirectional MR, and CellOracle-based GRN/co-expression analyses. The final output distinguishes consistently supported tissue-level candidates from exploratory candidates.

**FIGURE 2:**
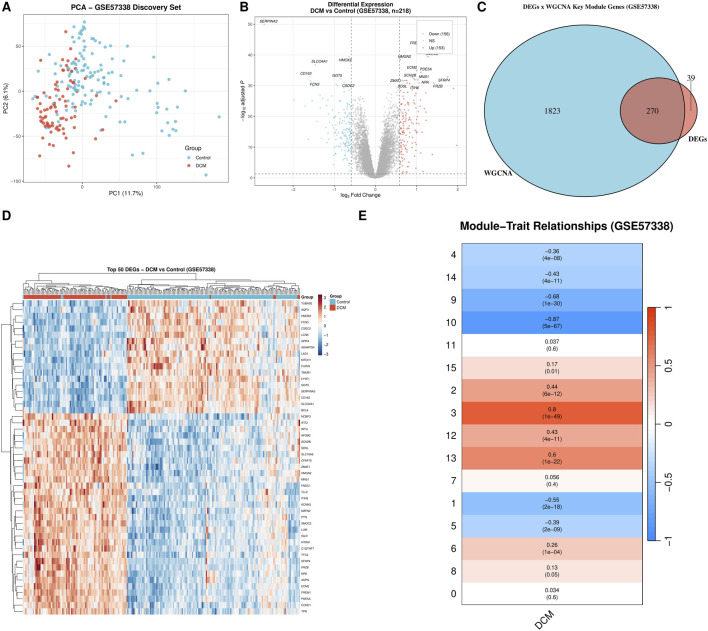
Identification of candidate genes through DEG and WGCNA integration. **(A)** PCA of the discovery cohort (GSE57338) demonstrating clear separation between DCM (n = 82) and control (n = 136) groups. **(B)** Volcano plot of 309 significant DEGs (red: upregulated; blue: downregulated; adjusted P < 0.05, |log_2_FC| > 0.585). **(C)** Venn diagram showing the intersection of 309 DEGs and 2,093 WGCNA hub genes, yielding 270 candidate genes. **(D)** Heatmap of the top 50 DEGs. **(E)** Module–trait correlation heatmap from WGCNA, with the purple module (r = −0.866) and brown module (r = +0.799) exhibiting the strongest associations with DCM status.

**FIGURE 3:**
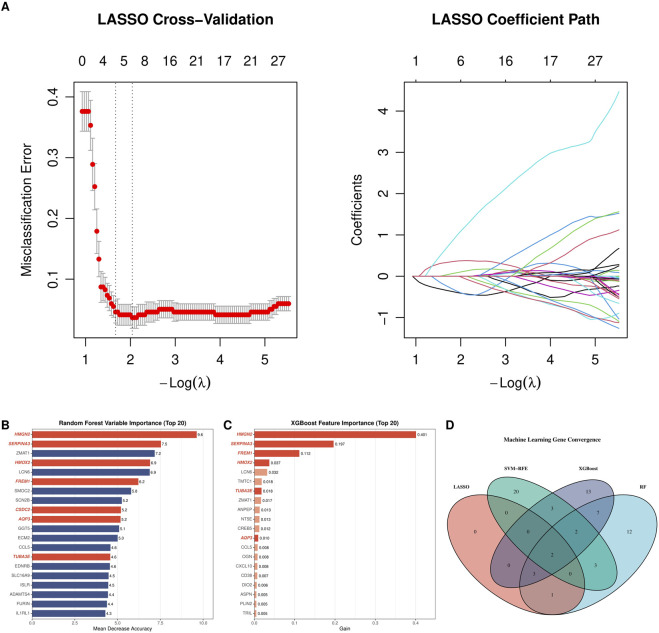
Machine learning-based identification of seven core diagnostic candidates. **(A)** LASSO coefficient path and 10-fold cross-validation curve. **(B)** Random forest variable importance (top 30 genes). **(C)** XGBoost feature importance (top 30 genes by gain). **(D)** Venn diagram of genes selected by four algorithms, with seven genes supported by ≥ 3 methods highlighted.

**FIGURE 4:**
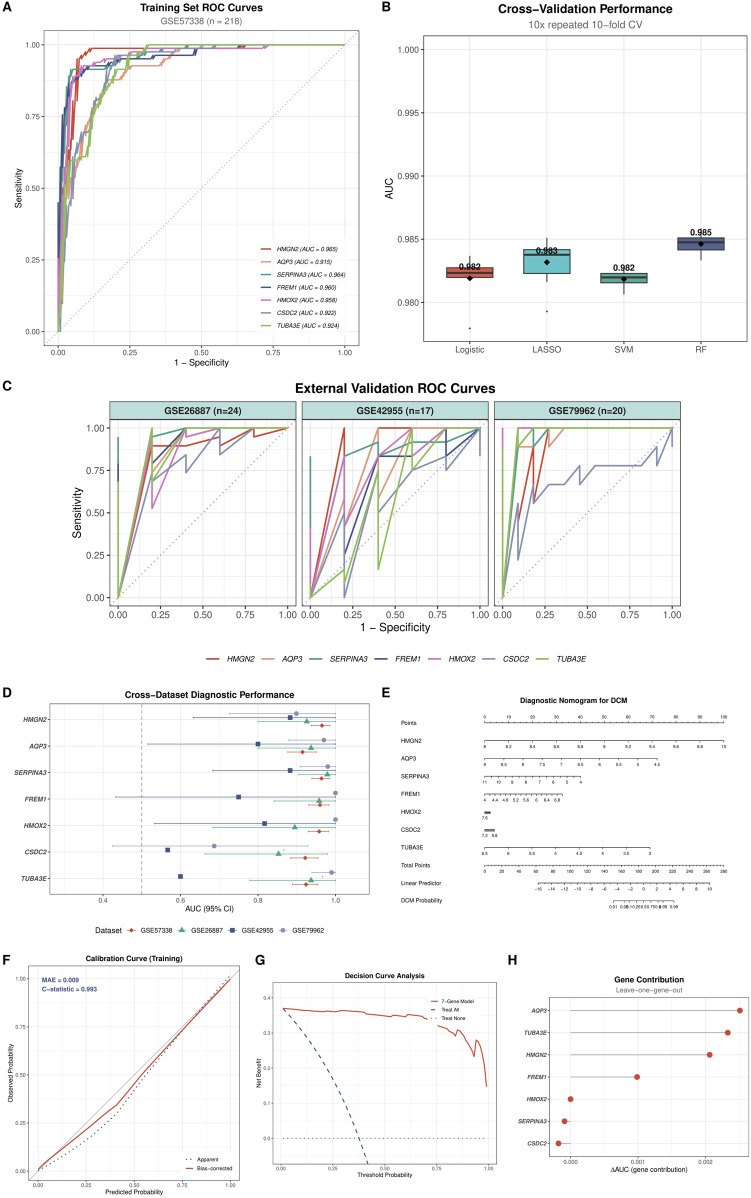
Tissue-level diagnostic model performance and gene contribution analysis. **(A)** ROC curves for individual candidates and the combined seven-gene panel in the discovery cohort. **(B)** Repeated cross-validation performance of four classifiers in the discovery cohort. Discovery-cohort performance estimates should be interpreted as apparent upper-bound estimates because feature selection was not nested within cross-validation. **(C,D)** External validation of individual candidate performance across independent microarray cohorts. **(E)** Nomogram visualizing tissue-level predictions from the logistic regression model, not a clinically deployable scoring tool. **(F)** Apparent calibration curve in the discovery cohort. **(G)** Decision curve analysis showing analytical net benefit across threshold probabilities. **(H)** Leave-one-gene-out analysis assessing the relative contribution of each candidate to model performance.

**FIGURE 5:**
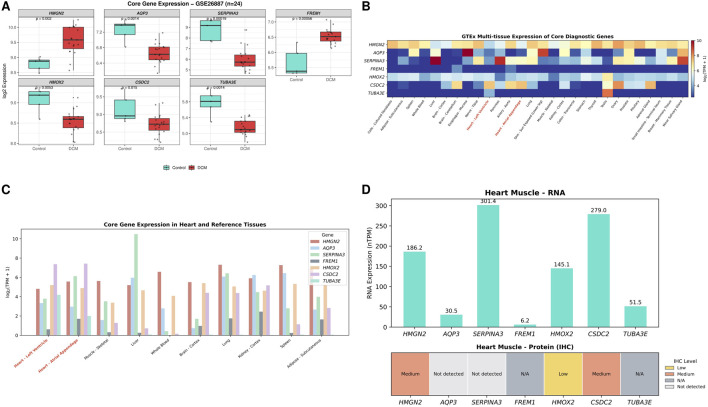
Multi-layered expression validation. **(A)** Expression box plots of seven core genes in the GSE26887 validation cohort (DCM vs. control). **(B)** GTEx v8 multi-tissue expression heatmap (log_2_ [TPM + 1]) across 25 tissues, with heart tissues highlighted in red. **(C)** GTEx expression in heart left ventricle, atrial appendage, and reference tissues. **(D)** HPA validation: RNA expression (nTPM) versus protein detection level (IHC) in heart muscle.

**FIGURE 6:**
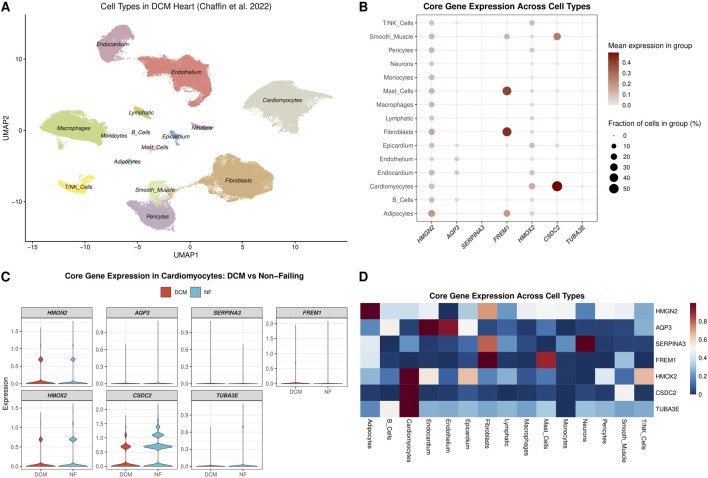
Single-nucleus RNA-seq validation using the Chaffin et al. (2022) DCM dataset. **(A)** UMAP visualization of 80,000 nuclei colored by 21 cell types (DCM + NF hearts). **(B)** Dot plot showing expression fraction and mean expression of seven core genes across cell types. **(C)** Violin plots comparing core gene expression between DCM and non-failing hearts in cardiomyocyte populations. **(D)** Matrix plot of standardized expression across all cell types.

**FIGURE 7:**
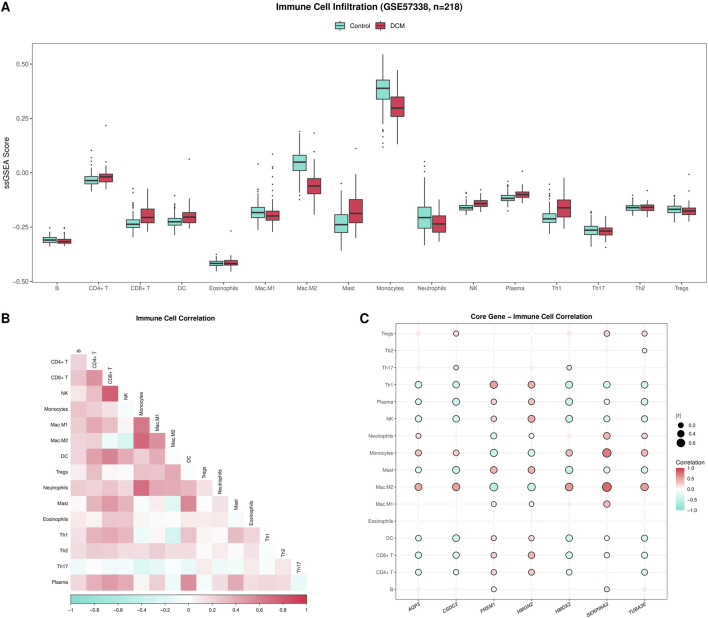
Immune microenvironment characterization. **(A)** Box plot comparing ssGSEA-derived immune cell abundances between DCM and control groups (16 cell types). **(B)** Immune cell–cell correlation heatmap. **(C)** Bubble plot of Spearman correlations between seven core genes and 16 immune cell types.

**FIGURE 8:**
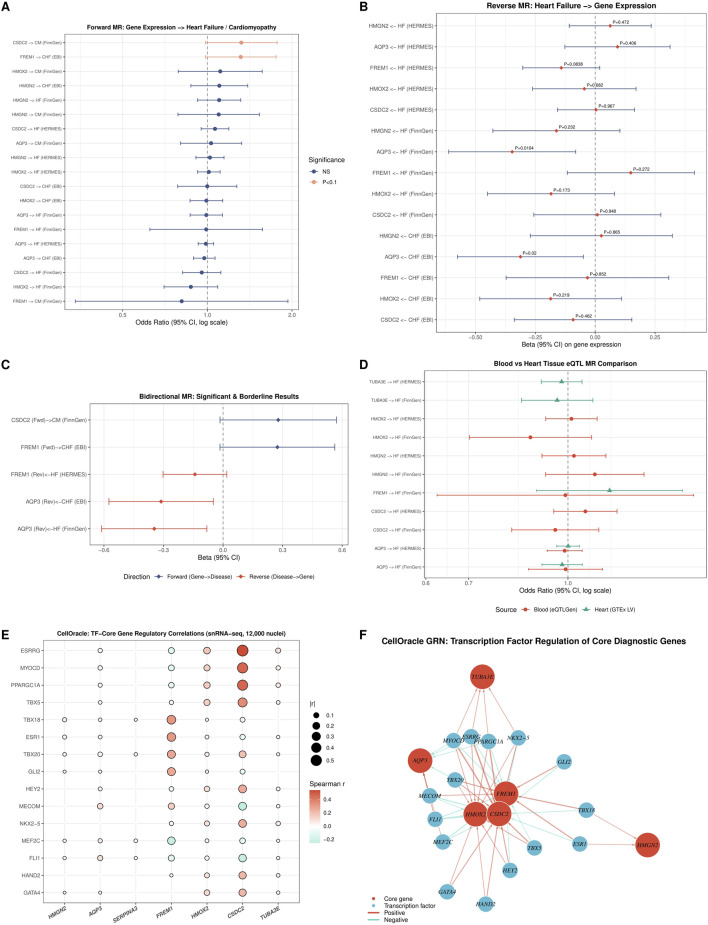
Exploratory MR and gene regulatory network analyses. **(A)** Forward MR forest plot evaluating genetically predicted candidate-gene expression in relation to heart failure or cardiomyopathy outcomes using blood-derived eQTL instruments. **(B)** Reverse MR forest plot evaluating heart failure-associated changes in candidate-gene expression; AQP3 showed a nominal replicated reverse-direction signal across two GWAS resources. **(C)** Bidirectional MR summary of nominal and non-significant signals. **(D)** Comparison of blood-derived and GTEx heart left ventricle eQTL-based MR estimates. **(E)** CellOracle-based TF–candidate correlation bubble plot; bubble size represents |Spearman r|, and color indicates correlation direction. PPARGC1A showed the strongest correlation with CSDC2. **(F)** GRN visualization showing putative TF–candidate associations inferred from snRNA-seq-derived GRN analysis. These MR and GRN analyses are hypothesis-generating and do not establish causal gene action or definitive regulatory hierarchy.

To clarify the interpretive strength of each analytical layer, the Results are organized into three tiers, as illustrated in Figure 1. Tier 1 covers discovery, feature selection, and tissue-level classification performance (Sections 3.1–3.3; Figures 2–4; Table 3). Tier 2 covers orthogonal localization evidence from GTEx, HPA, and snRNA-seq (Sections 3.4, 3.5; Figures 5, 6; Table 4). Tier 3 covers immune profiling, bidirectional MR, and CellOracle-based GRN and co-expression analyses (Sections 3.6 and 3.8, 3.9; Figures 7, 8; Table 5), which are interpreted as hypothesis-generating rather than causal evidence. Within this framework, SERPINA3, HMOX2, FREM1, and HMGN2 are treated as consistently supported candidates, whereas AQP3, CSDC2, and TUBA3E are treated as exploratory candidates. Table 6 summarizes the cumulative evidence for each gene.

### 3.1 Identification of candidate genes through DEG and WGCNA integration

Principal component analysis of the GSE57338 discovery cohort showed clear separation between DCM (n = 82) and control (n = 136) samples, supporting its suitability for differential expression analysis (Figure 2A). A total of 309 significant DEGs were identified, including 153 upregulated and 156 downregulated genes, using an adjusted P-value < 0.05 and |log_2_FC| > 0.585 as thresholds (Figure 2B). Among these, SERPINA3 showed the largest absolute fold change (log_2_FC = −2.698, adjusted P = 6.44 × 10^−55^), followed by FREM1 (log_2_FC = 1.390, adjusted P = 5.26 × 10^−48^) and HMOX2 (log_2_FC = −0.864, adjusted P = 3.47 × 10^−46^).

WGCNA of the top 5,000 variable genes identified 16 co-expression modules (Supplementary Figures S1A,B). Ten modules showed significant correlations with DCM status (|r| > 0.3, P < 0.05), with the purple module (r = −0.866, P = 4.82 × 10^−67^) and brown module (r = +0.799, P = 1.48 × 10^−49^) showing the strongest associations (Figure 2E). From these significant modules, 2,093 hub genes were extracted using MM > 0.5 and GS > 0.15. Intersecting the 309 DEGs with these WGCNA hub genes yielded 270 candidate genes for downstream machine-learning feature selection (Figure 2C).

### 3.2 Machine learning convergence identifies seven core diagnostic candidates

Four machine learning algorithms were applied to the 270 candidate genes (Figures 3A–C). LASSO regression selected six non-zero coefficient genes, whereas RF, SVM-RFE, and XGBoost each identified the top 30 genes according to their respective importance metrics. Intersection analysis identified seven genes supported by at least three of the four algorithms: HMGN2 and AQP3 were selected by all four algorithms, while SERPINA3, FREM1, HMOX2, CSDC2, and TUBA3E were selected by three algorithms (Figure 3D). Four of these genes—SERPINA3, FREM1, HMOX2, and TUBA3E—also ranked among the top 10 DEGs, supporting partial convergence between differential expression magnitude and machine-learning feature prioritization. The characteristics of the seven core diagnostic candidates, including differential expression statistics, algorithm support, and diagnostic performance, are summarized in Table 2.

**TABLE 2:** Summary of seven core diagnostic candidates identified by machine-learning convergence.

Gene	log_2_FC	Adjusted P	Direction	ML algorithms (n/4)	Discovery AUC	Mean validation AUC
*HMGN2*	0.604	3.45 × 10^−39^	Up	4/4	0.965	0.903
*AQP3*	−1.059	2.20 × 10^−27^	Down	4/4	0.915	0.902
*SERPINA3*	−2.698	1.20 × 10^−50^	Down	3/4	0.964	0.947
*FREM1*	1.076	1.12 × 10^−45^	Up	3/4	0.960	0.903
*HMOX2*	−0.639	4.08 × 10^−40^	Down	3/4	0.958	0.904
*CSDC2*	−0.760	9.17 × 10^−32^	Down	3/4	0.922	0.702
*TUBA3E*	−0.919	1.19 × 10^−30^	Down	3/4	0.924	0.842

log_2_FC, log_2_ fold change (DCM, vs. Control) in the discovery set (GSE57338). Adjusted P, Benjamini–Hochberg corrected P-value. ML, algorithms, number of machine learning methods (out of LASSO, RF, SVM-RFE, XGBoost) that selected this gene. Discovery AUC, area under the ROC, curve in GSE57338. Mean Validation AUC, average AUC, across three external validation cohorts (GSE26887, GSE42955, GSE79962). Genes supported by ≥ 3 of 4 algorithms were defined as core diagnostic candidates.

### 3.3 Diagnostic model performance and gene contribution analysis

The seven-gene combined model showed high apparent discriminative performance in the discovery cohort under 10 repeats of 10-fold cross-validation: RF achieved the highest mean AUC (0.985; 95% CI: 0.983–0.987), followed by LASSO (0.983), logistic regression (0.982), and SVM (0.982) (Figures 4A,B). Because feature selection was not nested within the cross-validation loop, these discovery-set estimates should be interpreted as potentially optimistic upper bounds. Individual gene ROC analysis showed that all seven candidates exceeded an AUC of 0.91 in the discovery cohort, with HMGN2 (0.965) and SERPINA3 (0.964) showing the strongest individual discrimination (Figure 4A).

External validation was performed across three independent microarray cohorts (Figures 4C,D). Because GSE26887 was annotated as heart failure rather than strictly DCM, its AUCs should be interpreted as discrimination between advanced heart failure and controls rather than DCM-specific validation. SERPINA3 showed the highest mean microarray validation AUC (0.947), followed by HMOX2 (0.904), HMGN2 (0.903), and FREM1 (0.903). In the small GSE79962 cohort, FREM1 and HMOX2 both reached an AUC of 1.000. All seven candidates showed AUC > 0.85 in GSE26887, whereas only five of seven maintained AUC > 0.75 in GSE42955, indicating uneven external robustness across the panel (Supplementary Figures S2A–C). Complete AUCs are shown in Table 3, and cumulative gene-level support is summarized in Table 6.

**TABLE 3:** Diagnostic performance (AUC) of seven core diagnostic candidates across discovery and validation cohorts.

Gene	GSE57338 (training)	GSE26887	GSE42955	GSE79962	Mean validation
*HMGN2*	**0.965** (0.937–0.986)	**0.926**	0.883	0.899	**0.903**
*AQP3*	**0.915** (0.877–0.950)	**0.937**	0.800	**0.970**	**0.902**
*SERPINA3*	**0.964** (0.939–0.984)	**0.979**	0.883	**0.980**	**0.947**
*FREM1*	**0.960** (0.931–0.983)	**0.958**	0.750	**1.000**	**0.903**
*HMOX2*	**0.958** (0.930–0.982)	0.895	0.817	**1.000**	**0.904**
*CSDC2*	**0.922** (0.884–0.955)	0.853	0.567	0.687	0.702
*TUBA3E*	**0.924** (0.889–0.954)	**0.937**	0.600	**0.990**	0.842

AUC, area under the receiver operating characteristic curve. All numeric values in the GSE57338 (training), GSE26887, GSE42955, GSE79962, and Mean validation columns are AUC point estimates. Values in parentheses in the GSE57338 (training) column indicate 95% confidence intervals from 2,000 stratified bootstrap replicates. Mean validation represents the arithmetic mean of AUC values across GSE26887, GSE42955, and GSE79962. Bold values indicate AUC ≥ 0.90.

The multivariate logistic regression model incorporating all seven candidates yielded a discovery-set C-statistic of 0.993 (Nagelkerke *R* ^2^ = 0.956). Because feature selection was not nested within cross-validation, this estimate should be interpreted as an upper bound of tissue-level discriminative performance. HMGN2 (P = 0.010) and AQP3 (P = 0.042) were independently associated with DCM status. The nomogram is presented as a visualization of tissue-level predictions rather than a clinically deployable scoring tool. Apparent calibration was close in the discovery cohort (MAE = 0.009; Figure 4F), and binned calibration was assessed in validation cohorts (Supplementary Figure S2D). DCA showed favourable analytical net benefit across threshold probabilities of 0.1–0.9 (Figure 4G; Supplementary Figure S2E), interpreted as analytical performance rather than clinical readiness.

LOOGO analysis showed that removing any single candidate produced only minimal AUC reduction in the discovery cohort (ΔAUC < 0.003), suggesting statistical complementarity within the seven-gene model (Figure 4H). Across the validation cohorts, removal of SERPINA3 produced the largest decrease in AUC, indicating its strongest apparent contribution to external validation performance (Supplementary Figure S2F).

Individual candidate performance was not uniform across validation cohorts. CSDC2 showed low AUCs in GSE42955 (0.567) and GSE79962 (0.687), approaching chance-level discrimination, while TUBA3E achieved an AUC of 0.600 in GSE42955. These inconsistencies may partly reflect the small validation sample sizes, which can produce wide confidence intervals and unstable point estimates. However, they also indicate that CSDC2 and TUBA3E may have limited standalone diagnostic value. Although the seven-gene model showed higher apparent discovery-set performance than any individual candidate, LOOGO analysis suggested that removing CSDC2 or TUBA3E had minimal effect on model performance (ΔAUC ≈ 0), raising the possibility that their role in the combined model is complementary or partly attributable to a free-rider effect rather than dominant predictive information (Supplementary Table S2).

### 3.4 Multi-layered expression validation

#### 3.4.1 Independent cohort validation

Box plot analysis showed concordant differential expression patterns of the seven core diagnostic candidates across the three microarray validation cohorts, with expression directions consistent with the discovery cohort (Figure 5A; Supplementary Figures S3A,B).

#### 3.4.2 RNA-seq cross-platform validation

To address the limitation of microarray-only validation, we further evaluated the candidates in GSE116250, an independent RNA-seq cohort comprising 37 DCM and 14 non-failing left ventricular samples. Five of seven candidates achieved AUC > 0.81: SERPINA3 (0.992), HMOX2 (0.971), FREM1 (0.963), HMGN2 (0.954), and TUBA3E (0.815). AQP3 showed moderate performance (0.699), whereas CSDC2 performed near chance level (0.535), consistent with its instability in the microarray validation cohorts. These RNA-seq results further support SERPINA3, HMOX2, FREM1, and HMGN2 as the most consistently validated tissue-level candidates.

#### 3.4.3 GTEx tissue expression

Analysis of GTEx v8 median TPM values across 54 human tissues showed that all seven candidates were expressed in cardiac tissue (Figure 5B). CSDC2 showed tissue-enhanced expression in heart muscle (300.6 nTPM), while HMOX2 (137.9 nTPM) and HMGN2 (186.2 nTPM) showed moderate-to-high cardiac expression. TUBA3E exhibited group-enriched expression in heart and testis (51.2 nTPM), supporting its cardiac relevance despite less stable diagnostic performance. Comparative analysis across heart left ventricle, atrial appendage, and reference tissues revealed distinct tissue-expression profiles for each candidate (Figure 5C).

#### 3.4.4 HPA protein expression

Human Protein Atlas immunohistochemistry detected protein expression in heart muscle for HMGN2 (Medium), CSDC2 (Medium), and HMOX2 (Low) (Figure 5D). SERPINA3 and AQP3 showed high RNA expression (301.4 and 30.5 nTPM, respectively) but were not detected at the protein level by IHC, a pattern consistent with SERPINA3 as a secreted acute-phase protein and AQP3 as a membrane channel with restricted epithelial localization. FREM1 and TUBA3E lacked completed IHC annotations in HPA v23. Together, these RNA–protein findings provide orthogonal context for interpreting the tissue-level relevance of the candidate markers. A summary of multi-layered expression evidence is presented in Table 4.

**TABLE 4:** Multi-layered expression validation of seven core diagnostic candidates.

Gene	GTEx heart LV (log_2_ [TPM+1])	HPA RNA (nTPM)	HPA protein (IHC)	snRNA-seq primary cell type	Heart eQTL
*HMGN2*	4.81	186.2	Medium	Broad (macrophage enriched)	Yes (F = 11.7)
*AQP3*	3.33	30.5	Not detected	Endothelial (sparse)	Yes (F = 111.9)
*SERPINA3*	3.79	301.4	Not detected	Fibroblast, neuronal	Yes (F = 15.7)
*FREM1*	0.62	6.2	N/A (pending)	Fibroblast (enriched)	Yes (F = 26.3)
*HMOX2*	5.20	145.1	Low	Cardiomyocyte (enriched)	Yes (F = 13.0)
*CSDC2*	7.36	279.0	Medium	Cardiomyocyte (enriched)	Yes (F = 16.7)
*TUBA3E*	4.19	51.5	N/A (inconclusive)	Low overall expression	No

GTEx, Heart LV, median expression in heart left ventricle from GTEx v8 (log_2_ [TPM+1] transformed). HPA RNA, consensus RNA, expression from the Human Protein Atlas (nTPM). HPA, Protein (IHC), immunohistochemistry-based protein detection level in heart muscle (High/Medium/Low/Not detected). snRNA-seq, Primary Cell Type, predominant expressing cell type from Chaffin et al. (2022) snRNA-seq, data (GSE183852). Heart eQTL, availability of significant cis-eQTL, in GTEx v8 heart left ventricle (F = F-statistic for instrument strength). N/A indicates data not available in HPA, v23.

### 3.5 Single-nucleus RNA sequencing-based cell-type localization

To resolve cell-type-specific expression patterns, we analyzed snRNA-seq data from 592,689 nuclei derived from human left ventricular tissue. After retaining DCM and non-failing samples and subsampling to 80,000 nuclei, UMAP visualization resolved 21 annotated cell populations, including Cardiomyocyte_I/II/III, Fibroblast_I/II, Endothelial_I/II/III, Pericyte_I/II, Macrophage, Lymphocyte, Mast cell, VSMC, Endocardial, Adipocyte, Neuronal, Lymphatic endothelial, and Epicardial cells (Figure 6A).

All seven core diagnostic candidates were detectable in the snRNA-seq dataset. Dot plot analysis showed distinct cell-type expression patterns (Figure 6B): HMOX2 and CSDC2 were mainly expressed in cardiomyocytes, with HMOX2 also detected in endocardial cells and pericytes. FREM1 was enriched in fibroblasts and activated fibroblasts, consistent with its extracellular matrix-related function. SERPINA3 was detected at low levels across multiple cell types, with relatively higher expression in fibroblasts and neuronal cells. AQP3 expression was sparse and mainly observed in endothelial subpopulations. HMGN2 showed broad expression across cell types, with enrichment in proliferating macrophages. TUBA3E showed low overall expression and limited cell-type specificity in the snRNA-seq data.

Violin plot analysis of cardiomyocyte populations from DCM and non-failing hearts showed differential expression patterns for several core diagnostic candidates (Figure 6C). Matrix plot visualization across cell types and disease conditions provided an integrated overview of the cell-type-resolved expression landscape (Figure 6D).

### 3.6 Immune microenvironment characterization

ssGSEA-based immune infiltration analysis showed significant differences in 13 of 16 immune cell types between DCM and control groups (FDR < 0.05) (Figure 7A). M2 macrophages showed the most pronounced decrease in DCM (FDR = 3.78 × 10^−19^), followed by monocytes (FDR = 1.55 × 10^−10^). Conversely, mast cells (FDR = 1.34 × 10^−8^), NK cells (FDR = 1.01 × 10^−10^), and Th1 cells (FDR = 1.94 × 10^−10^) were increased in DCM, suggesting a shift toward a more pro-inflammatory immune profile (Figure 7B). Results for all 16 immune cell types are provided in Supplementary Table S1.

Spearman correlation analysis showed associations between core diagnostic candidate expression and immune cell abundance, with distinct correlation patterns across genes (Figure 7C). Sensitivity analysis using an independent marker-gene scoring approach showed 100% directional concordance with the ssGSEA results among the 11 evaluable cell types, supporting the consistency of the immune-infiltration findings across analytical methods. Together, these results suggest that the candidate panel may capture immune-related transcriptional variation in DCM, although these associations remain correlative.

### 3.7 Functional enrichment analysis

GO biological process analysis of the 309 DEGs identified 1,230 enriched terms (adjusted P < 0.05), with extracellular matrix organization, collagen fibril organization, muscle contraction, and inflammatory response among the top categories (Supplementary Figure S1C). KEGG pathway analysis identified 127 enriched pathways, including cardiac muscle contraction, dilated cardiomyopathy, ECM–receptor interaction, and NF-κB signaling (Supplementary Figure S1D). GSEA identified 62 pathways at nominal P < 0.1, with focal adhesion and ECM–receptor interaction enriched in DCM, whereas oxidative phosphorylation and cardiac muscle contraction were relatively depleted in DCM (Supplementary Figure S1E).

### 3.8 Bidirectional Mendelian randomization and tissue-specific eQTL analysis

Of the seven core diagnostic candidates, five (HMGN2, AQP3, HMOX2, CSDC2, and FREM1) had available eQTLGen instruments with F-statistics > 10. SERPINA3 and TUBA3E were excluded from blood eQTL-based MR because genome-wide significant cis-eQTL instruments were unavailable.

#### 3.8.1 Forward MR (gene expression → heart failure)

Using blood-derived eQTLs, no candidate showed a statistically significant forward association with heart failure at P < 0.05. CSDC2 showed a nominal association with cardiomyopathy that did not reach statistical significance (OR = 1.321, 95% CI: 0.985–1.771, P = 0.063), while HMOX2 showed a non-significant protective direction (OR = 0.874, 95% CI: 0.702–1.088, P = 0.229) (Figure 8A). Overall, the forward MR results did not support a causal role for these candidates in heart failure development.

#### 3.8.2 Reverse MR (heart failure → gene expression)

AQP3 was the only candidate with a nominally significant reverse-direction signal. Heart failure in FinnGen was associated with lower AQP3 expression (β = −0.347, SE = 0.135, P = 0.010), and a similar signal was observed using the EBI chronic heart failure GWAS (IVW: β = −0.312, SE = 0.134, P = 0.020; weighted median: P = 0.016) (Figure 8B; Supplementary Figures S4A,D). Given the limited number of instruments and nominal P-value thresholds, this finding should be interpreted as hypothesis-generating evidence that AQP3 may be disease-responsive rather than as definitive causal evidence. The complete bidirectional MR results are presented in Table 5.

**TABLE 5:** Bidirectional Mendelian randomization results for core diagnostic candidates.

Gene	Direction	Outcome/Exposure	Method	nSNP	Or/Beta	95% CI	P value
*HMGN2*	Forward	HF (FinnGen)	Wald ratio	1	OR = 1.101	0.923–1.314	0.285
*AQP3*	Forward	HF (HERMES)	Wald ratio	1	OR = 0.988	0.928–1.052	0.712
*HMOX2*	Forward	HF (FinnGen)	IVW	2	OR = 0.874	0.702–1.088	0.229
*CSDC2*	Forward	CM (FinnGen)	IVW	2	OR = 1.321	0.985–1.771	0.063
*FREM1*	Forward	HF (FinnGen)	Wald ratio	1	OR = 0.991	0.625–1.571	0.968
*AQP3*	Reverse	HF (FinnGen)	Wald ratio	1	β = −0.347	—	0.010*
*AQP3*	Reverse	CHF (EBI)	IVW	3	β = −0.312	—	0.020*
*HMOX2*	Reverse	HF (FinnGen)	Wald ratio	1	β = −0.184	—	0.173
*HMGN2*	Reverse	HF (FinnGen)	Wald ratio	1	β = −0.162	—	0.232
*CSDC2*	Reverse	HF (HERMES)	IVW	7	β = 0.003	—	0.967

Forward MR, gene expression → heart failure/cardiomyopathy (eQTLGen, blood eQTL, as exposure). Reverse MR, heart failure → gene expression (GWAS, as exposure, eQTLGen eQTL, as outcome). OR, odds ratio (forward direction). β, effect estimate on gene expression scale (reverse direction). IVW, inverse variance weighted method; HF, heart failure; CM, cardiomyopathy; CHF, chronic heart failure. *P < 0.05, statistically significant. SERPINA3 and TUBA3E were excluded due to absence of genome-wide significant cis-eQTL, variants in eQTLGen.

#### 3.8.3 Cardiac tissue-specific MR

Using GTEx heart left ventricle eQTLs, six of seven candidates, all except TUBA3E, had cardiac cis-eQTL instruments with F-statistics > 10. Cardiac eQTL-based MR showed broadly similar effect directions to blood eQTL-based MR, but no forward association reached statistical significance, likely reflecting the limited number of available instruments (Figure 8D; Supplementary Figure S4E). SERPINA3 showed the most suggestive cardiac eQTL-based association with heart failure (OR = 1.130, P = 0.169 for HERMES), a signal not observed using blood eQTLs. These comparisons suggest that tissue context may influence MR estimates, although the results remain exploratory. Full cardiac tissue-specific MR results are provided in Supplementary Table S3.

### 3.9 Gene regulatory network and putative upstream TF context

Gene co-expression analysis of the snRNA-seq data showed pairwise correlations among the core diagnostic candidates (Figure 8E). The strongest positive correlation was observed between HMOX2 and CSDC2 (Spearman r = 0.310, P < 2.2 × 10^−16^), whereas FREM1 and CSDC2 showed the strongest negative correlation (r = −0.123). These co-expression patterns were integrated with STRING PPI data to construct an interaction network.

CellOracle-based GRN inference was performed across 21 cell-type clusters using ridge regression and the human promoter base GRN. Because the seven candidates are not transcription factors, they could not be directly used as perturbation inputs in CellOracle’s TF-centered simulation framework. We therefore used the inferred GRN and expression correlations to identify putative upstream TFs associated with the candidate genes. PPARGC1A (PGC-1α) showed the strongest correlation with CSDC2 (r = 0.598) and HMOX2 (r = 0.326), while MECOM (EVI1) was correlated with FREM1 (r = 0.205) and inversely correlated with CSDC2 (r = −0.251). ID1 was correlated with AQP3 (r = 0.160), consistent with its known role in endothelial differentiation. These findings provide hypothesis-generating regulatory context for the candidate genes rather than evidence of a definitive transcriptional hierarchy.

The co-expression-based functional importance score was used as an author-defined descriptive ranking of gene–cell type pairs (Figure 8F). Because this score has not been experimentally validated or benchmarked against perturbation datasets, these rankings should be interpreted only as exploratory prioritization signals. CSDC2 showed the highest score in Cardiomyocyte_I cells (score = 0.417), FREM1 in fibroblast populations (score = 0.386), and HMOX2 showed relatively broad scores across multiple cell types. These patterns are consistent with the cell-type expression profiles and suggest that the candidate panel may reflect transcriptomic variation across multiple cardiac cell compartments (Supplementary Table S4).

### 3.10 Gene-by-layer evidence synthesis

To summarize cumulative evidence across analytical layers, we compiled a gene-by-layer synthesis in Table 6. SERPINA3, HMOX2, FREM1, and HMGN2 met the Tier 1 external-validation criterion (defined as mean microarray validation AUC ≥ 0.90 and RNA-seq AUC ≥ 0.95) and were supported by orthogonal localization evidence; they were therefore classified as consistently supported candidates. AQP3 showed less uniform external performance but contributed the only nominal replicated reverse-direction MR signal, supporting its classification as an exploratory candidate of mechanistic interest. CSDC2 and TUBA3E showed unstable validation performance in at least one small external cohort and were classified as exploratory complementary candidates requiring further confirmation. This classification is maintained throughout the manuscript.

**TABLE 6:** Gene-by-layer evidence synthesis and tier classification for the seven candidate diagnostic genes.

Gene	Tier 1 — discovery (log_2_FC; adj. P; ML n/4; CV AUC)	Tier 1 — external validation (microarray mean AUC/range; RNA-seq GSE116250 AUC)	Tier 2 — GTEx heart RNA/HPA protein in cardiac muscle	Tier 2 — snRNA-seq dominant cell type	Tier 3 — MR and GRN signal	Tier classification
*SERPINA3*	log_2_FC = −2.698; adj. P = 1.2 × 10^−50^; ML 3/4; CV AUC = 0.964	Microarray mean AUC = 0.947 (0.883–0.980); RNA-seq AUC = 0.992	High RNA in heart; IHC not detected (secreted protein)	Fibroblast, neuronal (low–moderate)	No eQTL for blood MR; suggestive cardiac-tissue MR signal (P = 0.169)	Tier 1, consistently supported
*HMOX2*	log_2_FC = −0.639; adj. P = 4.1 × 10^−40^; ML 3/4; CV AUC = 0.958	Microarray mean AUC = 0.904 (0.817–1.000); RNA-seq AUC = 0.971	Medium RNA; IHC low	Cardiomyocyte	Non-significant MR; GRN upstream PPARGC1A (r = 0.326)	Consistently supported candidate
*FREM1*	log_2_FC = +1.076; adj. P = 1.1 × 10^−45^; ML 3/4; CV AUC = 0.960	Microarray mean AUC = 0.903 (0.750–1.000); RNA-seq AUC = 0.963	Low RNA; IHC pending	Fibroblast, activated fibroblast	Non-significant MR; GRN upstream MECOM (r = 0.205)	Consistently supported candidate
*HMGN2*	log_2_FC = +0.604; adj. P = 3.5 × 10^−39^; ML 4/4; CV AUC = 0.965	Microarray mean AUC = 0.903 (0.883–0.926); RNA-seq AUC = 0.954	Medium RNA; IHC medium	Broad expression	Non-significant MR	Consistently supported candidate
*AQP3*	log_2_FC = −1.059; adj. P = 2.2 × 10^−27^; ML 4/4; CV AUC = 0.915	Microarray mean AUC = 0.902 (0.800–0.970); RNA-seq AUC = 0.699	Low RNA; IHC not detected (epithelial-restricted)	Endothelial (sparse)	Reverse MR P = 0.010 (FinnGen)/0.020 (EBI CHF); GRN upstream ID1	Exploratory candidate — mechanistic interest
*CSDC2*	log_2_FC = −0.760; adj. P = 9.2 × 10^−32^; ML 3/4; CV AUC = 0.922	Microarray mean AUC = 0.702 (0.567–0.853); RNA-seq AUC = 0.535	High RNA; IHC medium	Cardiomyocyte	Non-significant MR; GRN upstream PPARGC1A (r = 0.598)	Exploratory complementary candidate
*TUBA3E*	log_2_FC = −0.919; adj. P = 1.2 × 10^−30^; ML 3/4; CV AUC = 0.924	Microarray mean AUC = 0.842 (0.600–0.990); RNA-seq AUC = 0.815	Medium RNA; IHC inconclusive	Low overall expression	No eQTL for MR analysis	Exploratory complementary candidate

AUC, values rounded to three decimals; Tier-1, external criterion = microarray mean AUC ≥ 0.90 and GSE116250 RNA-seq AUC ≥ 0.95. MR, findings reaching nominal P < 0.05 are shown in bold. Microarray validation cohorts: GSE26887, GSE42955, GSE79962. Single-cell cell-type assignments reflect dominant expression in the Chaffin et al. (2022) snRNA-seq, dataset (GSE183852). HPA, status refers to immunohistochemistry in heart muscle in HPA, v23. Genes are listed in tier order: SERPINA3, HMOX2, FREM1, and HMGN2 are classified as Tier 1 consistently supported candidates; AQP3 is classified as Tier 2 exploratory (mechanistic interest); CSDC2 and TUBA3E are classified as Tier 2 exploratory (complementary).

## 4 Discussion

In this study, we developed a multi-layer bioinformatic discovery and prioritization pipeline that nominates seven tissue-level candidate biomarkers for DCM—SERPINA3, HMOX2, FREM1, HMGN2, AQP3, CSDC2, and TUBA3E—and places them within an explicit hierarchy of evidence. Beyond multi-cohort validation, the pipeline incorporates orthogonal localization evidence from GTEx, HPA immunohistochemistry, and single-nucleus RNA sequencing, together with hypothesis-generating mechanistic context from immune profiling, bidirectional MR using blood-derived and cardiac tissue-specific eQTLs, and CellOracle-based GRN analysis with co-expression-based functional importance scoring.

To avoid assigning equivalent weight to all analytical components, we discuss the findings according to three evidence levels: tissue-level diagnostic performance (Tier 1), cellular localization evidence from GTEx, HPA, and snRNA-seq (Tier 2), and hypothesis-generating causal/regulatory context from MR and GRN analyses (Tier 3). Discovery-set performance is interpreted as an upper bound because feature selection was not nested within cross-validation, while MR and GRN/functional-importance-score results are treated as exploratory rather than causal. Accordingly, we distinguish consistently supported candidates—SERPINA3, HMOX2, FREM1, and HMGN2—from exploratory candidates—AQP3, CSDC2, and TUBA3E—throughout the Discussion.

### 4.1 Tissue-level classification performance and candidate prioritization

At the level of primary evidence, the seven-gene panel showed strong apparent discriminative performance in myocardial tissue. The RF classifier achieved an AUC of 0.985 under repeated cross-validation, and the logistic model yielded a discovery-set C-statistic of 0.993 with close apparent calibration. However, because DEG filtering, WGCNA hub extraction, and machine-learning convergence were performed before cross-validation, these discovery-set metrics may be optimistic and are best interpreted as upper-bound estimates. The nomogram should therefore be regarded as a visualization of tissue-level model predictions, and DCA as analytical net benefit, rather than evidence of clinical readiness.

External validation clarified that the seven candidates did not have equal support. SERPINA3, HMOX2, FREM1, and HMGN2 showed the most consistent performance across external microarray cohorts and the independent RNA-seq cohort GSE116250, and are therefore treated as the most consistently supported candidates. SERPINA3 showed the strongest single-gene support, with stable cross-dataset discrimination and the largest apparent LOOGO contribution. As a serine protease inhibitor and acute-phase protein, SERPINA3 has been implicated in cardiovascular inflammation, myocardial remodeling, and extracellular-matrix homeostasis (Lok et al., 2011). HMOX2 is involved in heme degradation and antioxidant defense, a process relevant to oxidative-stress injury in heart failure (Abraham and Kappas, 2008; Tsutsui et al., 2011). FREM1 encodes an extracellular-matrix protein related to basement-membrane integrity, consistent with its fibroblast-enriched expression and ECM-remodeling context (Smyth and Scambler, 2005; Frangogiannis, 2019). HMGN2, a nucleosome-binding protein involved in chromatin accessibility, may reflect transcriptional and epigenetic remodeling in DCM (Postnikov and Bustin, 2010; Haldar and McKinsey, 2014).

In contrast, AQP3, CSDC2, and TUBA3E are better interpreted as exploratory candidates. AQP3 showed less uniform diagnostic performance but contributed the only nominal replicated reverse-direction MR signal, suggesting possible disease responsiveness. Its known role as a water and glycerol channel also supports further investigation in endothelial or fluid-homeostasis contexts (Hara-Chikuma and Verkman, 2006). CSDC2 showed cardiomyocyte-enriched expression but unstable external validation, including near-chance performance in GSE42955 and GSE116250. As a cold-shock-domain protein involved in post-transcriptional RNA regulation, it remains biologically interesting but not yet a reliable single-gene classifier (Graumann and Marahiel, 1998). TUBA3E also showed variable validation performance and lacked suitable cis-eQTL instruments for MR analysis. These exploratory candidates may provide complementary biological coverage within the panel, but their individual predictive value requires confirmation in larger and better-phenotyped DCM cohorts.

### 4.2 Orthogonal localization evidence from GTEx, HPA, and snRNA-seq

GTEx, HPA, and snRNA-seq provided complementary rather than redundant evidence because they address different questions. GTEx assessed tissue-level cardiac expression, HPA evaluated RNA–protein concordance in heart muscle, and snRNA-seq resolved the cell types contributing to the bulk myocardial signal (GTEx Consortium, 2020; Chaffin et al., 2022; Uhlén et al., 2015). Together, these layers helped contextualize the tissue-level candidates.

For HMOX2 and CSDC2, GTEx and snRNA-seq supported cardiomyocyte-related expression, with HPA providing additional protein-level support (GTEx Consortium, 2020; Chaffin et al., 2022; Uhlén et al., 2015). FREM1 showed fibroblast-enriched expression, consistent with extracellular-matrix biology (Smyth and Scambler, 2005; Frangogiannis, 2019). SERPINA3 showed high cardiac RNA but absent local IHC detection, compatible with its biology as a secreted acute-phase protein and suggesting potential relevance for future circulating-biomarker studies (Lok et al., 2011). AQP3 showed sparse endothelial localization, consistent with its known channel biology and supporting its exploratory classification (Hara-Chikuma and Verkman, 2006).

These localization findings support biological plausibility but do not establish functional validity, disease causation, or clinical applicability. They should be interpreted as orthogonal context for candidate prioritization rather than as experimental validation. This interpretation is consistent with previous single-nucleus transcriptomic evidence showing broad cell-type-specific remodeling in DCM hearts (Chaffin et al., 2022).

### 4.3 Immune microenvironment in DCM

Immune profiling showed differences in 13 of 16 immune cell types between DCM and control samples, with M2 macrophages showing the most pronounced decrease. M2 macrophages are involved in tissue repair, fibrosis resolution, and anti-inflammatory signaling (Epelman et al., 2015), and their reduction may be consistent with impaired reparative responses in DCM. The concomitant increase in mast cells, NK cells, and Th1 cells suggests a shift toward a more pro-inflammatory immune profile, consistent with the broader inflammatory cardiomyopathy paradigm (Seferović et al., 2019; Heymans et al., 2009; Patella et al., 1998).

Correlations between candidate-gene expression and immune-cell abundance further suggest that the panel may capture immune-related transcriptional variation in DCM. However, these results are computational and correlative, and should not be interpreted as evidence that the candidate genes directly regulate immune infiltration.

### 4.4 Mendelian randomization: exploratory genetic evidence

The MR analyses should be interpreted as exploratory genetic evidence rather than definitive causal inference, given the limited number of instruments and nominal P-value thresholds. In forward MR, no candidate showed a statistically significant effect on heart failure, and the nominal association between CSDC2 and cardiomyopathy did not reach significance. This is particularly important because MR can help distinguish potential causal relationships from associations, but its reliability depends strongly on instrument availability and validity (Davey Smith and Hemani, 2014).

In reverse MR, AQP3 showed the only nominal replicated signal, with heart failure associated with lower AQP3 expression in both FinnGen and EBI chronic heart failure datasets. This pattern is consistent with possible heart failure-associated downregulation of AQP3, but it does not establish strong causality. AQP3 is therefore best interpreted as a potential disease-responsive candidate rather than a confirmed causal driver of DCM.

Cardiac tissue-specific MR provided an additional tissue-aware check using GTEx heart left ventricle eQTLs (GTEx Consortium, 2020). Six candidates had available cardiac cis-eQTL instruments, but no forward association reached statistical significance. The broadly similar directions between blood- and cardiac-tissue MR estimates support biological plausibility, whereas limited instrument availability and modest GTEx heart sample size constrain causal interpretation. The suggestive cardiac-tissue signal for SERPINA3 should therefore be viewed as a weak tissue-level hypothesis requiring further validation, not as evidence of SERPINA3–heart failure causality.

Taken together, the MR results indicate that the seven candidates should be interpreted primarily as tissue-level disease-responsive markers of DCM-associated myocardial remodeling rather than confirmed disease-driving genes. This interpretation is consistent with the diagnostic and prioritization purpose of the present study, but it does not support therapeutic-target claims.

### 4.5 Gene regulatory network and upstream transcriptional context

CellOracle-based GRN analysis provided hypothesis-generating regulatory context for the candidate genes (Kamimoto et al., 2023). Because the seven candidates are not transcription factors, they could not be directly used as perturbation inputs in CellOracle’s TF-centered simulation framework. Therefore, the inferred TF–gene relationships should be interpreted as correlative regulatory context rather than formal perturbation evidence.

PPARGC1A (PGC-1α) showed the strongest correlation with the cardiomyocyte-enriched candidates CSDC2 and HMOX2. PGC-1α is a major regulator of cardiac energy metabolism and mitochondrial biogenesis, and its downregulation is a recognized feature of DCM and heart failure (Arany et al., 2005). The observed correlations are consistent with a model in which reduced PGC-1α activity may be associated with altered CSDC2 and HMOX2 expression in DCM myocardium, but this model requires direct experimental testing. Similarly, MECOM showed a correlative association with FREM1, and ID1 with AQP3, consistent with their known biological contexts but not establishing direct regulation (Frangogiannis, 2019; Hara-Chikuma and Verkman, 2006).

The co-expression-based functional importance score should be viewed as an author-defined descriptive ranking of gene–cell type pairs, not as a perturbation simulation or gene-ablation model. The ranking highlighted CSDC2 in cardiomyocytes, FREM1 in fibroblasts, and HMOX2 across multiple cell types, generating testable hypotheses for future functional studies. Because this metric has not been benchmarked against CRISPR screens, RNAi experiments, or established perturbation outputs, its predictive accuracy is unknown (Graumann and Marahiel, 1998). These results do not provide causal evidence on their own.

### 4.6 Translational considerations and tissue accessibility

A key translational limitation is that the model was derived from myocardial gene expression profiles. Direct myocardial transcriptomic profiling would require endomyocardial biopsy, which is invasive and subject to sampling limitations (Schultheiss et al., 2019). Therefore, this study does not provide a clinically deployable diagnostic test. Its main contribution is the prioritization of tissue-level molecular candidates for future translational work.

Several directions may help move these candidates closer to clinical relevance, but their feasibility is uneven. SERPINA3 currently has the clearest translational priority because it encodes a secreted acute-phase protein, showed the strongest single-gene support, and may be measurable in peripheral blood (Lok et al., 2011). Its high cardiac RNA expression but absent local IHC detection is compatible with secretion into the circulation, supporting dedicated blood-based protein validation. By contrast, the feasibility of translating the other six candidates into circulating biomarkers is uncertain, particularly for nuclear or intracellular proteins such as HMGN2 and CSDC2. cfRNA profiling and exosome-mediated RNA transport may provide exploratory routes for detecting cardiac-derived transcripts in peripheral blood, but these approaches remain speculative until directly validated (Koh et al., 2014; Huang et al., 2019). Thus, the present panel should be viewed as a prioritized set of tissue-level candidates for liquid-biopsy, proteomic, and experimental validation, not as a clinical test in its current form.

### 4.7 Methodological strengths and limitations

The main strength of this study is its tiered integrative design. At the diagnostic level, the four-algorithm ML convergence strategy reduced dependence on a single feature-selection method, and validation across three microarray cohorts plus an independent RNA-seq cohort provided external support. At the localization level, GTEx, HPA, and snRNA-seq addressed tissue expression, RNA–protein concordance, and cell-type localization (GTEx Consortium, 2020; Chaffin et al., 2022; Uhlén et al., 2015). At the exploratory mechanistic level, MR and GRN/functional-importance-score analyses generated testable hypotheses, although their evidentiary strength is lower than that of the external classification analyses (Davey Smith and Hemani, 2014; Kamimoto et al., 2023).

Several limitations should be acknowledged. First, the discovery and microarray validation datasets were derived from Affymetrix platforms, although GSE116250 provided independent RNA-seq validation; additional large-scale RNA-seq cohorts would further strengthen the findings. Second, feature selection was not nested within cross-validation; therefore, discovery-set performance estimates should be interpreted as upper bounds despite external validation. Third, GSE26887 was annotated as heart failure rather than strictly DCM, introducing phenotypic heterogeneity. Fourth, MR analyses were constrained by limited genetic instruments, and the non-significant forward MR results suggest that these candidates should not be interpreted as confirmed disease-driving genes or therapeutic targets. Fifth, the co-expression-based functional importance score is an author-defined descriptive metric that has not been benchmarked against CRISPR screens, RNAi experiments, or validated perturbation outputs; its predictive accuracy is therefore unknown. Sixth, the model is based on myocardial tissue expression, limiting immediate clinical applicability. Seventh, the translational feasibility of most candidates as circulating biomarkers remains uncertain, except for SERPINA3, which has the clearest rationale because of its secretory nature. Finally, *in vitro* and *in vivo* experimental validation was not performed.

Future studies should validate these candidates in larger, phenotypically well-defined DCM cohorts, incorporate additional RNA-seq and prospective datasets, evaluate simplified gene panels focused on consistently validated candidates, perform blood-based protein/cfRNA/exosomal validation—especially for SERPINA3—and conduct dedicated experimental perturbation studies to test the mechanistic hypotheses generated here.

## 5 Conclusion

We developed a multi-layer bioinformatic discovery and prioritization framework that identified seven tissue-level, predominantly disease-responsive candidate biomarkers for DCM. Four candidates—SERPINA3, HMOX2, FREM1, and HMGN2—showed the most consistent support across external microarray cohorts and the independent RNA-seq cohort GSE116250, whereas AQP3, CSDC2, and TUBA3E showed less uniform external performance and should be considered exploratory. Although the discovery-set models showed high apparent performance, these estimates should be interpreted as upper bounds because feature selection was not nested within cross-validation. Orthogonal evidence from GTEx, HPA, and snRNA-seq supported tissue-level expression and cell-type localization, while MR and GRN analyses generated hypothesis-generating signals, including possible heart failure-associated AQP3 downregulation and a putative PPARGC1A–CSDC2/HMOX2 regulatory context. Overall, these findings provide a prioritized set of disease-responsive tissue-level molecular candidates and testable hypotheses for future translational investigation in DCM, rather than disease-driving therapeutic targets or a clinically deployable diagnostic test.

## Data Availability

The original contributions presented in the study are included in the article/Supplementary Material, further inquiries can be directed to the corresponding author.
