# Supplementary material for: Identification and multi-layered validation of seven diagnostic biomarkers for dilated cardiomyopathy via integrative machine learning, single-cell transcriptomics, and Mendelian randomization
Source: Front Cell Dev Biol. 2026 Jun 9;14:1851275. doi: 10.3389/fcell.2026.1851275 (PMC13286953; doi:10.3389/fcell.2026.1851275)
Supplement: Supplementary file 1 [file Supplementaryfile1.docx]

**Supplementary Tables：**

**Table S1. Immune cell infiltration differences between DCM and control groups (ssGSEA).**

| **Immune Cell Type** | **DCM (Mean Score)** | **Control (Mean Score)** | **Difference** | **P-value** | **FDR** |
| --- | --- | --- | --- | --- | --- |
| Macrophages M2 | −0.055 | 0.043 | −0.098 | 2.36 × 10⁻²⁰ | 3.78 × 10⁻¹⁹ |
| NK cells | −0.140 | −0.160 | 0.019 | 1.26 × 10⁻¹¹ | 1.01 × 10⁻¹⁰ |
| Monocytes | 0.301 | 0.378 | −0.077 | 2.91 × 10⁻¹¹ | 1.55 × 10⁻¹⁰ |
| Th1 cells | −0.161 | −0.205 | 0.044 | 4.85 × 10⁻¹¹ | 1.94 × 10⁻¹⁰ |
| CD8+ T cells | −0.197 | −0.232 | 0.035 | 4.64 × 10⁻⁹ | 1.34 × 10⁻⁸ |
| Mast cells | −0.175 | −0.232 | 0.057 | 5.02 × 10⁻⁹ | 1.34 × 10⁻⁸ |
| Plasma cells | −0.100 | −0.115 | 0.015 | 1.61 × 10⁻⁷ | 3.69 × 10⁻⁷ |
| Dendritic cells | −0.200 | −0.223 | 0.023 | 2.46 × 10⁻⁶ | 4.92 × 10⁻⁶ |
| Neutrophils | −0.234 | −0.200 | −0.034 | 0.00209 | 0.00344 |
| CD4+ T cells | −0.019 | −0.031 | 0.012 | 0.00215 | 0.00344 |
| Macrophages M1 | −0.185 | −0.179 | −0.006 | 0.00705 | 0.01026 |
| B cells | −0.313 | −0.309 | −0.004 | 0.01162 | 0.01549 |
| Tregs | −0.174 | −0.168 | −0.006 | 0.01325 | 0.01630 |
| Th17 cells | −0.268 | −0.264 | −0.004 | 0.309 | 0.348 |
| Eosinophils | −0.413 | −0.417 | 0.004 | 0.327 | 0.348 |
| Th2 cells | −0.160 | −0.160 | 0.001 | 0.654 | 0.654 |

*Note: Immune cell abundance was estimated using ssGSEA (single-sample gene set enrichment analysis) with the GSVA package. P-values were calculated by Wilcoxon rank-sum test. FDR, false discovery rate (Benjamini–Hochberg correction). Rows are ordered by FDR. 13 of 16 immune cell types showed significant differences (FDR < 0.05) between DCM (n = 82) and control (n = 136) groups in the discovery cohort (GSE57338).*

**Table S2. Leave-one-gene-out (LOOGO) analysis of the diagnostic model.**

| **Model** | **Training AUC** | **GSE26887 AUC** | **GSE42955 AUC** | **GSE79962 AUC** | **ΔAUC (Training)** | **ΔAUC (Mean Val.)** |
| --- | --- | --- | --- | --- | --- | --- |
| Full (7 genes) | 0.993 | 1.000 | 0.950 | 1.000 | — | — |
| w/o HMGN2 | 0.991 | 1.000 | 0.950 | 1.000 | 0.002 | 0.000 |
| w/o AQP3 | 0.990 | 1.000 | 0.950 | 1.000 | 0.003 | 0.000 |
| w/o SERPINA3 | 0.993 | 0.989 | 0.933 | 1.000 | −0.000 | 0.009 |
| w/o FREM1 | 0.992 | 1.000 | 0.950 | 1.000 | 0.001 | 0.000 |
| w/o HMOX2 | 0.993 | 1.000 | 0.950 | 1.000 | 0.000 | 0.000 |
| w/o CSDC2 | 0.993 | 1.000 | 0.950 | 1.000 | −0.000 | 0.000 |
| w/o TUBA3E | 0.990 | 1.000 | 0.967 | 1.000 | 0.002 | −0.006 |

*Note: LOOGO, leave-one-gene-out analysis. For each core gene, a reduced 6-gene logistic regression model was trained on the discovery set and evaluated on all cohorts. ΔAUC = AUC(Full) − AUC(Reduced); positive values indicate performance decrease upon gene removal. ΔAUC (Mean Val.) is the mean ΔAUC across three validation cohorts. SERPINA3 removal produced the largest mean validation AUC decrease (0.009), indicating it is the single most important gene for external generalizability.*

**Table S3. Cardiac tissue-specific Mendelian randomization results (GTEx heart left ventricle eQTL).**

| **Gene** | **Outcome** | **Method** | **nSNP** | **OR** | **95% CI** | **P-value** | **F-statistic** |
| --- | --- | --- | --- | --- | --- | --- | --- |
| *HMGN2* | HF (HERMES) | Wald ratio | 1 | 0.909 | 0.768–1.077 | 0.270 | 11.7 |
| *AQP3* | HF (HERMES) | Wald ratio | 1 | 1.001 | 0.961–1.042 | 0.976 | 111.9 |
| *AQP3* | HF (FinnGen) | Wald ratio | 1 | 0.979 | 0.911–1.053 | 0.571 | 111.9 |
| *SERPINA3* | HF (HERMES) | Wald ratio | 1 | 1.130 | 0.949–1.347 | 0.169 | 15.7 |
| *SERPINA3* | HF (FinnGen) | Wald ratio | 1 | 0.813 | 0.604–1.095 | 0.174 | 15.7 |
| *FREM1* | HF (FinnGen) | Wald ratio | 1 | 1.161 | 0.893–1.510 | 0.263 | 26.3 |
| *HMOX2* | HF (FinnGen) | Wald ratio | 1 | 1.090 | 0.744–1.599 | 0.658 | 13.0 |
| *CSDC2* | HF (HERMES) | Wald ratio | 1 | 1.036 | 0.884–1.214 | 0.661 | 16.7 |
| *CSDC2* | HF (FinnGen) | Wald ratio | 1 | 0.936 | 0.711–1.234 | 0.641 | 16.7 |
| *TUBA3E* | — | — | — | — | — | No heart eQTL | — |

*Note: Cardiac tissue-specific MR using lead cis-eQTL variants from GTEx v8 heart left ventricle (n = 386 samples). OR, odds ratio per SD increase in genetically predicted gene expression. F-statistic assesses instrument strength (F > 10 indicates adequate strength). All instruments met the F > 10 threshold. TUBA3E had no significant cis-eQTL in GTEx heart left ventricle. HF, heart failure. SERPINA3 showed the most suggestive effect (OR = 1.130, P = 0.169 for HERMES), a signal not detected using blood eQTLs.*

**Table S4. Table S4. Co-expression-based functional importance score: top-ranked cell types per candidate gene.**

| **Gene** | **Top 1 Cell Type (Score)** | **Top 2 Cell Type (Score)** | **Top 3 Cell Type (Score)** | **Total Score (All Cell Types)** |
| --- | --- | --- | --- | --- |
| *CSDC2* | Cardiomyocyte_I (0.417) | Cardiomyocyte_II (0.356) | Pericyte_I (0.298) | High |
| *FREM1* | Fibroblast_I (0.386) | Activated_fibro. (0.329) | Pericyte_II (0.245) | High |
| *HMOX2* | Cardiomyocyte_I (0.371) | Endocardial (0.312) | Pericyte_I (0.289) | Highest |
| *HMGN2* | Macrophage (0.285) | Lymphocyte (0.241) | Endothelial_I (0.198) | Moderate |
| *SERPINA3* | Fibroblast_I (0.267) | Neuronal (0.223) | Epicardial (0.189) | Moderate |
| *AQP3* | Endothelial_II (0.198) | Endothelial_I (0.176) | Lymphatic_endo. (0.134) | Low |
| *TUBA3E* | Cardiomyocyte_I (0.156) | Pericyte_I (0.112) | Fibroblast_I (0.098) | Low |

*Note: The co-expression-based functional importance score was calculated as mean expression × fraction of expressing cells × summed absolute co-expression with the remaining core candidates. Higher scores indicate stronger co-expression-based prioritization of a given gene–cell type pair. This author-defined score is descriptive and should not be interpreted as a perturbation simulation, virtual knockout, gene-ablation effect, or causal prediction. Its predictive accuracy has not been benchmarked against CRISPR screens, RNAi experiments, or validated perturbation outputs. Genes are ordered by the total co-expression-based score.*
