# Supplementary figures and images for: Identification and multi-layered validation of seven diagnostic biomarkers for dilated cardiomyopathy via integrative machine learning, single-cell transcriptomics, and Mendelian randomization
Source: Front Cell Dev Biol. 2026 Jun 9;14:1851275. doi: 10.3389/fcell.2026.1851275 (PMC13286953; doi:10.3389/fcell.2026.1851275)

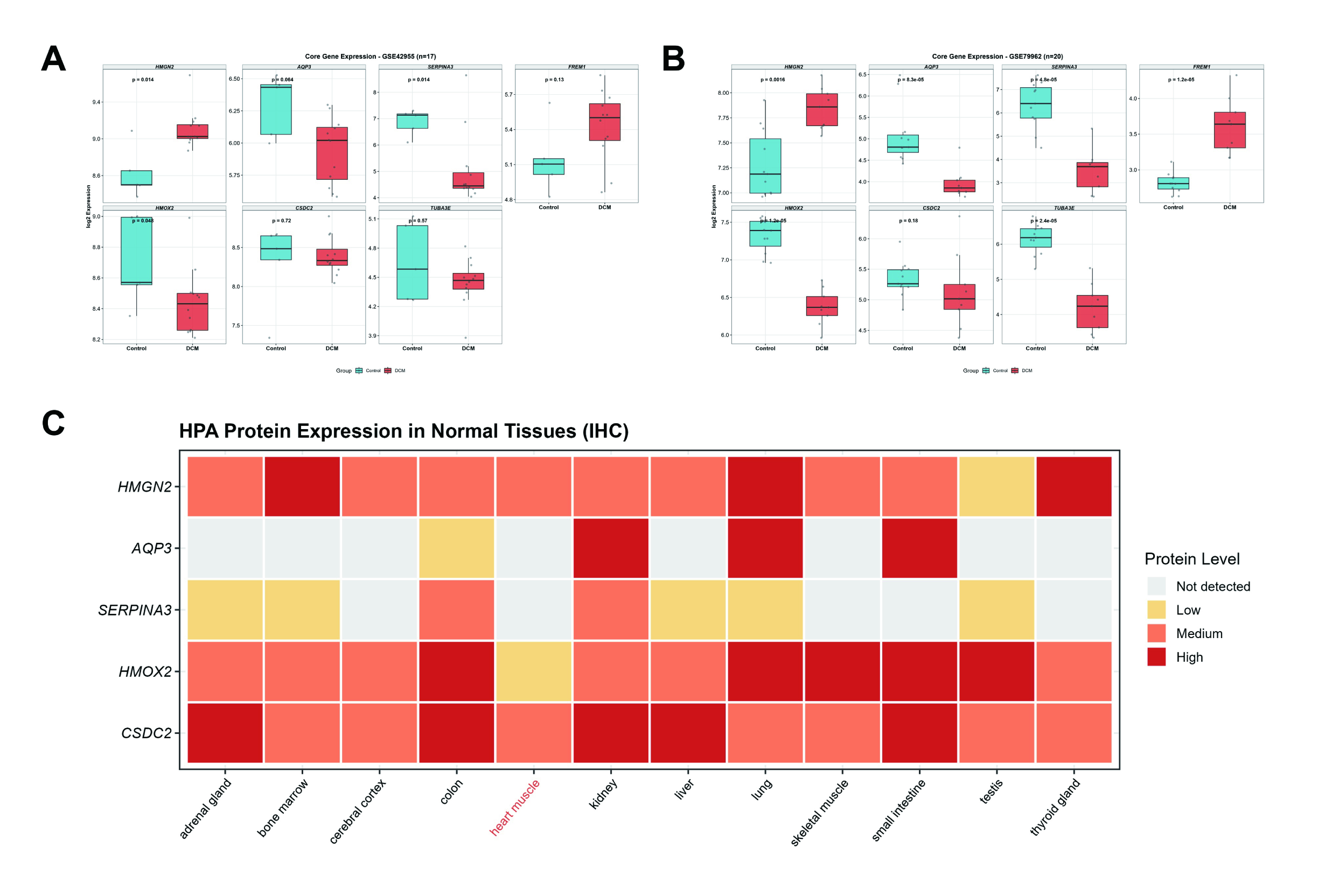

Supplement: Supplementary file 2 [file Image3.tif]

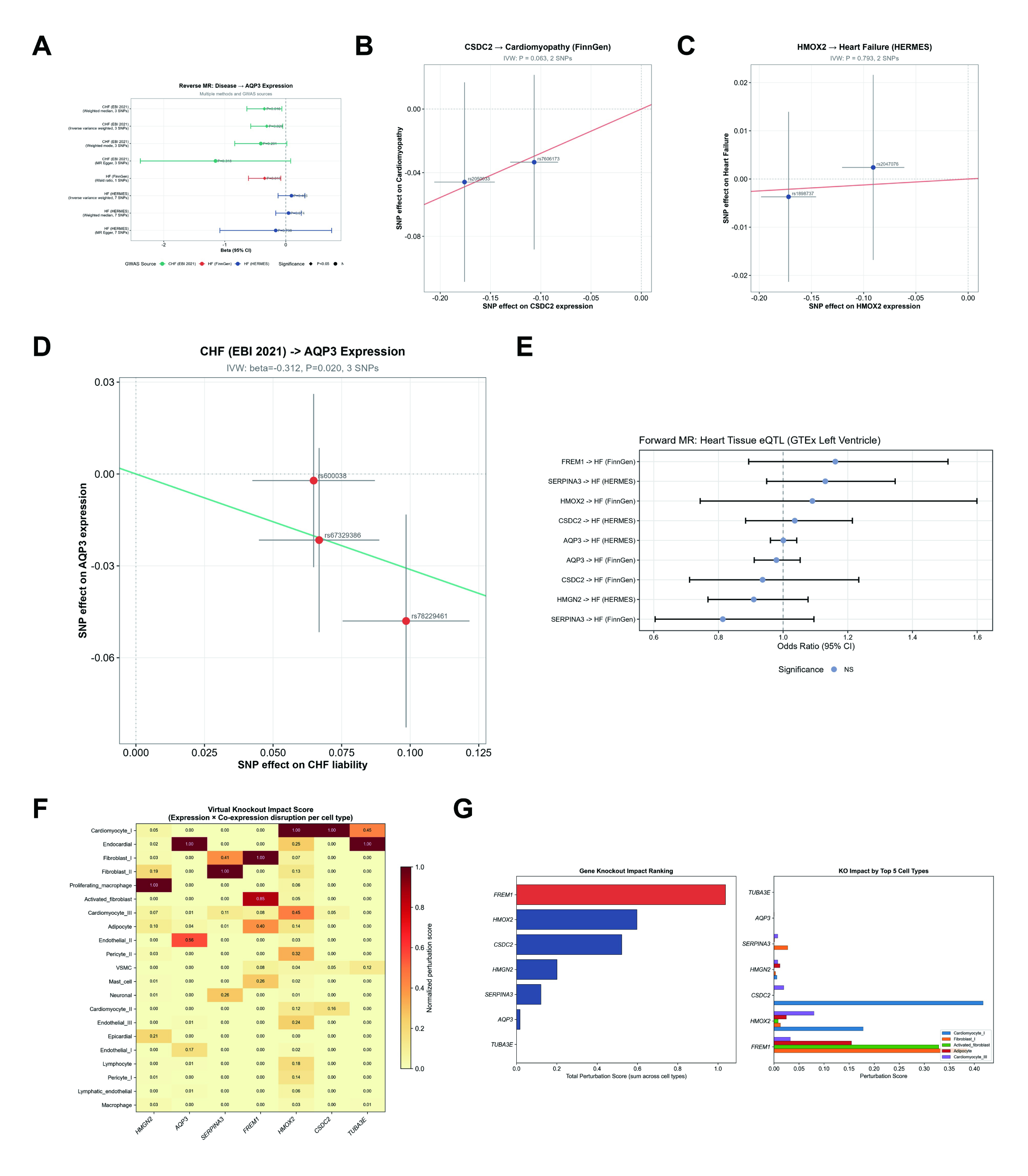

Supplement: Supplementary file 3 [file Image4.tif]

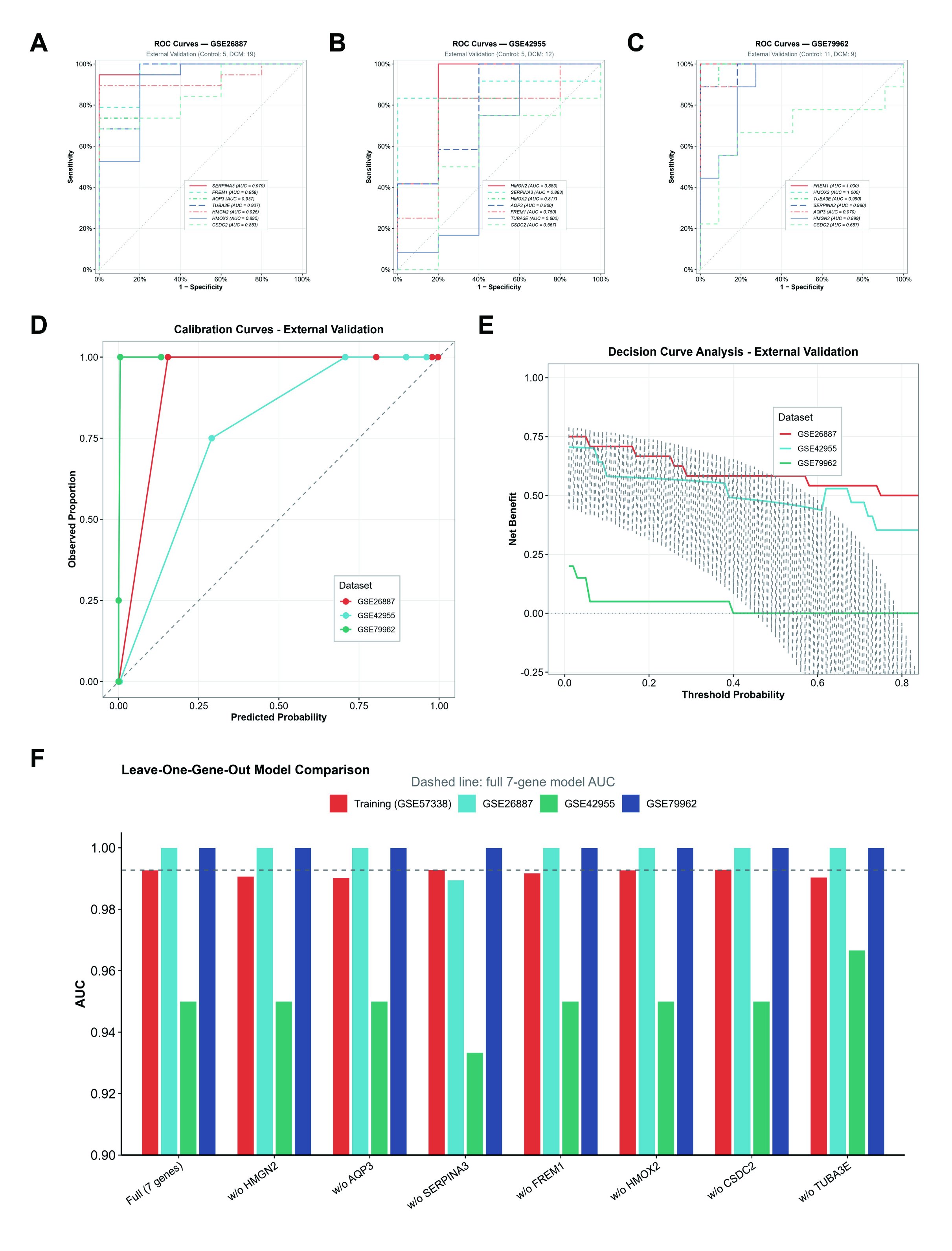

Supplement: Supplementary file 4 [file Image2.tif]

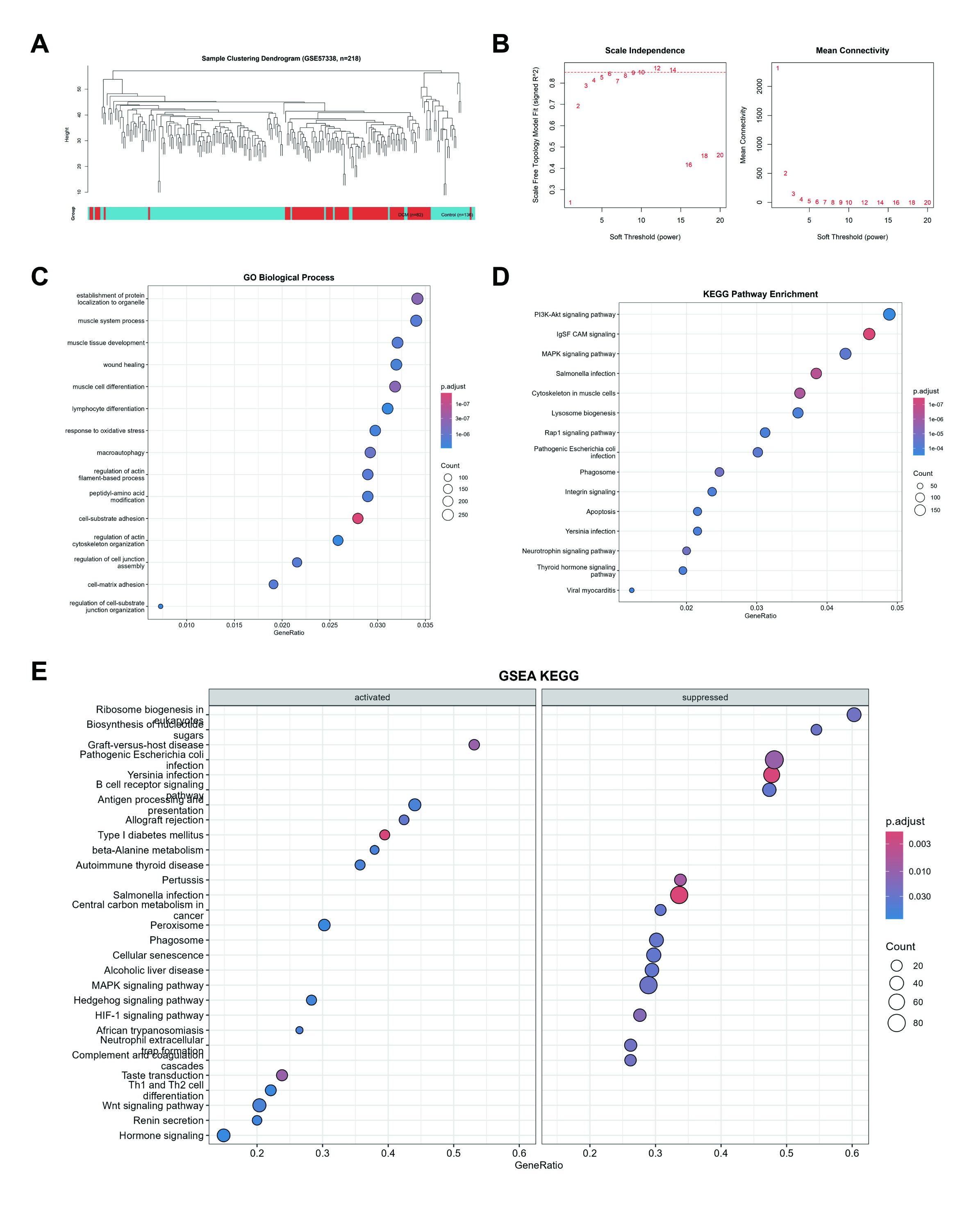

Supplement: Supplementary file 5 [file Image1.tif]
